# Approach to Strain Selection and the Propagation of Viral Stocks for Venezuelan Equine Encephalitis Virus Vaccine Efficacy Testing under the Animal Rule

**DOI:** 10.3390/v11090807

**Published:** 2019-08-31

**Authors:** Janice M. Rusnak, Pamela J. Glass, Scott C. Weaver, Carol L. Sabourin, Andrew M. Glenn, William Klimstra, Christopher S. Badorrek, Farooq Nasar, Lucy A. Ward

**Affiliations:** 1 Joint Program Executive Office for Chemical, Biological, Radiological and Nuclear Defense (JPEO-CBRND), Joint Project Manager-Medical Countermeasure Systems (JMP-MCS), Joint Vaccine Acquisition Program (JVAP), 1564 Freedman Drive, Fort Detrick, MD 21702, USA; 2 Department of Virology, United States Army Medical Research Institute of Infectious Diseases (USAMRIID), 1425 Porter Street, Fort Detrick, MD 21702, USA; 3 Institute for Human Infections and Immunity, World Reference Center for Emerging Viruses and Arboviruses and Department of Microbiology and Immunology, University of Texas Medical Branch, 301 University Boulevard, Galveston, TX 77555, USA; 4 Battelle Biomedical Research Center, 1425 Plain City-Georgesville Road, West Jefferson, OH 43162, USA; 5 Center for Vaccine Research, University of Pittsburgh, 3501 Fifth Avenue, Pittsburgh, PA 15261, USA

**Keywords:** Venezuelan equine encephalitis virus, vaccine, strain selection, Animal Rule, cDNA cloned virus, virus stock propagation

## Abstract

Licensure of a vaccine to protect against aerosolized Venezuelan equine encephalitis virus (VEEV) requires use of the U.S. Food and Drug Administration (FDA) Animal Rule to assess vaccine efficacy as human studies are not feasible or ethical. An approach to selecting VEEV challenge strains for use under the Animal Rule was developed, taking into account Department of Defense (DOD) vaccine requirements, FDA Animal Rule guidelines, strain availability, and lessons learned from the generation of filovirus challenge agents within the Filovirus Animal Nonclinical Group (FANG). Initial down-selection to VEEV IAB and IC epizootic varieties was based on the DOD objective for vaccine protection in a bioterrorism event. The subsequent down-selection of VEEV IAB and IC isolates was based on isolate availability, origin, virulence, culture and animal passage history, known disease progression in animal models, relevancy to human disease, and ability to generate sufficient challenge material. Methods for the propagation of viral stocks (use of uncloned (wild-type), plaque-cloned, versus cDNA-cloned virus) to minimize variability in the potency of the resulting challenge materials were also reviewed. The presented processes for VEEV strain selection and the propagation of viral stocks may serve as a template for animal model development product testing under the Animal Rule to other viral vaccine programs. This manuscript is based on the culmination of work presented at the “Alphavirus Workshop” organized and hosted by the Joint Vaccine Acquisition Program (JVAP) on 15 December 2014 at Fort Detrick, Maryland, USA.

## 1. Introduction

Venezuelan equine encephalitis (VEE) is a mosquito-borne illness, endemic in areas of South America, Central America, Mexico, Florida and Trinidad, that primarily affects equids and humans [1,2,3]. VEE disease in humans is typically manifested as an acute self-limiting febrile illness of 3 to 5 days duration, with an abrupt onset of fever and chills, severe headache, malaise, myalgia, and nausea. Unlike equids, encephalitis and mortality in humans from VEEV infection is uncommon (< 1% of cases) and is observed mainly in children and the elderly [4,5,6]. 

VEE virus (VEEV) has been designated a Category B biothreat agent as aerosol exposure to as few as 10 to 100 infectious particles results in symptomatic disease in nearly all humans. Aerosol-acquired VEE does not occur naturally. Thereby, as human efficacy studies are not ethical or feasible, licensure in the U.S. of a vaccine to protect against aerosol VEEV exposure must use the Food and Drug Administration (FDA) Animal Rule. The Animal Rule, as set forth in 21 Code of Federal Regulations Part 601.90–95, requires demonstration of vaccine protection in an animal model that is predictive of the response expected in humans. Vaccine efficacy demonstrated in animal models and the immune response to the vaccine observed in humans will be used to predict the likely clinical benefit in humans. 

Guidance for virus strain selection for the animal model in the FDA document “Product Development under the Animal Rule- Guidance for Industry” notes selection of the virus strain should consider strain origin, virulence, passage history in cultures and animals, known disease progression in the animal model, and relevancy to human disease [7]. The strain must also result in similar disease and pathophysiology in the selected animal model as occurs in humans by the designated exposure route. Logistical issues (e.g., strain availability, replication characteristics to generate challenge material) and the sponsor’s objectives for vaccine protection must also be considered in strain selection.

Selection criteria for VEEV isolates in the Department of Defense (DOD) Joint Vaccine Acquisition Program (JVAP) were reviewed, as well as the strategy in selecting the optimal methodology for the propagation of viral stocks to result in the consistency of the production of VEEV challenge material and reproducibility of animal challenge studies. The criteria for strain selection and strategy for the propagation of viral stocks may serve as a template for other viral vaccine products being developed under the Animal Rule. Lastly, current information and literature on VEEV aerosol challenge studies in animals with the selected strains was reviewed in regards to disease progression and pathophysiology in the animal models and the relevance of the selected strains and animal models to disease in humans. 

## 2. Background 

### 2.1. Alphaviral Committee and Workshop

Based on the recommendation of the FDA Office of Counter-Terrorism and Emergency Coordination Staff in 2012, a committee was organized by the JVAP alphavirus vaccine program to select VEEV strain(s) for animal model development under the Animal Rule, and to obtain concurrence on the strain selection by the FDA Center for Biologics Evaluation and Research (CBER) before developing the master and working viral stocks. The committee was comprised of representatives from JVAP, the U.S. Army Medical Research Institute of Infectious Diseases (USAMRIID), U.S. Army Medical Materiel Development Activity (USAMMDA), and Defense Threat Reduction Agency (DTRA), and involved consultation with multiple VEEV experts and institutes with virus repositories. An “Alphavirus Workshop” was then organized and hosted by the Joint Vaccine Acquisition Program (JVAP) at Fort Detrick, Maryland, on 15 December 2014 to present and discuss the activities of the alphavirus committee members and to address outstanding issues on the generation of VEEV challenge materials.

### 2.2. Strain Selection

The list of VEEV strains classified under the epizootic varieties (associated with equine-amplified epidemics) and enzootic varieties (maintained in rodent-mosquito cycles and generally not equine amplification-competent) of VEEV subtype I (VEEV IAB, IC, ID, and IE) was comprised after consultation with multiple institutes with known VEEV repositories, and did not include former VEE complex subtypes II–VI and VEEV IF due to their reclassification as distinct species that are now referred to by their prototype strain [8]. Relevant data for each strain included the source of the isolate, passage history, and country/year of initial isolation. 

Initial strain selection criteria had to consider the DOD vaccine requirement for vaccine protection against VEEV in an aerosolized bioterrorism event. Further down-selection considered the FDA’s guidance for strain selection under the Animal Rule (FDA 2015), which recommended the use of 1) strains isolated from lethal human cases (or associated with human disease/outbreaks), 2) strains with a known and low passage history in animals and cell culture, and 3) strains that mirrored the expected disease state in humans. Strain selection also considered criteria used by the Filovirus Animal Non-clinical Group (FANG) to select virus strains for the filovirus challenge stocks under the Animal Rule—to select isolates with no animal passage (if available) and limited cell passage (Table 1) [9]. Logistical issues considered included selection of strains that were available and accessible to laboratories licensed to work with select agents and strains that could be replicated to produce sufficient challenge material for animal models. 

### 2.3. Approach to Selecting a Methodology for the Propagation of Challenge Material

A workshop comprised of alphavirus experts from government, industry, and academia was organized to discuss options for generating viral challenge material that would minimize or avoid variation in the potency of challenge material across multiple sites. Advantages and disadvantages of the propagation of challenge material from uncloned (wild-type), plaque-cloned, versus cDNA-cloned virus were reviewed, and variation in plaque sizes observed in cell cultures of uncloned (wild-type) VEEV was addressed [10,11,12,13,14].

### 2.4. Comparison of VEE in Humans and Animal Models

Literature and existing studies in mice and nonhuman primates (NHPs) challenged with the two selected VEEV strains were reviewed. Pathophysiological mechanisms of virulence by exposure route and exposure dose in humans and in animal models were compared with regards to time to disease onset, manifestations of disease, morbidity and mortality, and pathophysiology. 

## 3. Summary of Workshop/Committee Proceedings

### 3.1. VEEV Strain Down-Selection for Animal Model

A total of 12 epizootic and 13 enzootic isolates were identified (Table 2). Down-selection to epidemic VEEV IAB and IC strains was based on the DOD objective to obtain FDA approval for a vaccine that protects against at least two VEEV varieties likely to be used in a bioterrorism event. These two epizootic varieties have been responsible for most epidemics and human cases, in contrast to endemic varieties (ID and IE) that are also associated with human infection but have not produced wide-spread epidemics [4,11,13,15]. In addition, VEEV subtype IAB was the only VEEV variety weaponized in the biological weapons programs of the U.S. and former Soviet Union, and thereby most likely to be involved in a bioterrorism event [16]. 

#### 3.1.1. VEEV IAB Strains

VEEV IAB strains were responsible for VEE epidemics and outbreaks from 1938 to 1973. Evidence suggested many VEE IAB outbreaks were related to immunization of animals with formalin-inactivated VEEV IAB vaccines containing residual live virus, as further VEE IAB outbreaks were not reported after discontinued use of the inactivated vaccines. However, continuous cryptic circulation as a source of epizootic emergence cannot be excluded [31]. 

Based on the selection criteria, isolates from two VEEV IAB strains were identified, VEEV Trinidad (TrD) and 69Z1 strains (Table 2). Other strains of VEEV IAB (e.g., human VEEV IAB E123 69 and E541 73 strains isolated in Venezuela in 1969 and 1973, respectively) were not considered as the viral stocks and primary sequences were not readily available. The VEEV 69Z1 human isolate from the 1969 Guatemala outbreak that was available at USAMRIID was unacceptable due to its poorly documented passage history [32,33,34,35]. However, a VEEV 69Z1 isolate available at University of Texas Medical Branch (UTMB) had a known and acceptable passage history, consisting of only two passages in suckling mice and one passage on Vero cells. 

The VEEV IAB TrD strain was originally isolated from brain tissue of an infected donkey in 1943 during the Trinidad outbreak [2,19,31,36]. As the Trinidad outbreak involved mainly equids, no TrD isolates from humans were available. Manifestation of disease in the 377 officially reported equid cases was most commonly a febrile illness accompanied by loss of appetite and depression, followed by neurological symptoms (e.g., somnolence, incoordination, muscle twitching/spasticity), and death (83% mortality reported but milder cases in rural areas may have been missed). Although not a human isolate, VEEV IAB TrD was extensively studied and weaponized in the U.S. and former Soviet Union weapons programs, and thereby was deemed a likely strain to be involved in a bioterrorism event. The VEEV TrD strain was also commonly used in animal studies to examine pathogenesis and to assess efficacy of vaccines and therapeutics [37,38,39,40,41,42,43,44,45,46,47,48]. However, most of the available VEEV TrD stocks had multiple passages in animal and culture cell lines since its early isolation in 1943 because adaptation of the virus in cell culture was often required to achieve the high viral titers needed for aerosol challenge of larger animal models [49,50]. In spite of the extensive passage history, several laboratories have demonstrated the continued virulence of these IAB VEEV stocks in animals and in humans, including infection in eight laboratory workers with the VEEV IAB Venezuela 1938 strain that had 52 passages in suckling mice [19,51,52,53]. Most TrD strains in the USAMRIID repository had a passage history of guinea pig—one, chick embroyo—14, suckling mouse brain—one, and an additional one or two passages in BHK cells (Table 2). However, one VEEV TrD stock had a lower passage history (guinea pig—one, chick embryo—13, duck embryo cells—one) and no passage history in BHK cells [17,18,19]. Lastly, the consensus sequence of a VEEV TrD stock (L01442.2) was available in the GenBank database and had a documented passage history of once in guinea pigs, six times in Vero cells, and once in BHK cells. This TrD stock was propagated by the CDC using the same original 1943 VEEV TrD donkey brain isolate as the USAMRIID stocks [21,22].

The VEEV TrD strain was selected for animal model development, even though isolated from donkey brain, based on its weaponization history and the extensive study data available. Also, documented cases of aerosol-acquired infection in humans due to VEEV TrD would allow for a comparison of aerosol-acquired disease in humans to the animal models (as required by the Animal Rule) [52]. The USAMRIID TrD stocks with documented passage histories in Table 2 were considered as options for use under the Animal Rule, as well as the TrD (L-01442.2) consensus sequence from the GenBank database. 

#### 3.1.2. VEEV IC Strains

The University of Texas Medical Branch (UTMB) had several available VEEV IC human isolates with a single passage in Vero cells, from which the INH-9813 and INH-6803 strains from the 1995 outbreak in Colombia and Venezuela were selected for further evaluation (Table 2). UTMB also had a VEEV IC SH3 strain (human isolate with a single passage in Vero cells) from a less extensive Venezuelan outbreak in 1993. Other VEEV IC strains listed in Table 2 were not considered as they were not human isolates, or because they had less favorable passage histories than the INH-9813 and INH-6803 human isolates [54]. 

The VEEV IC INH-9813 and INH-6803 strains from the more extensive 1995 VEE outbreak were selected for further evaluation. The evaluation included preparation and characterization of a master stock (passage 2) and working stock (passage 3) for each strain in American Type Culture Collection (ATCC) Vero E6 cells (CRL-1586). Deep sequence analysis demonstrated that the sequence of each isolate was consistent with a VEEV IC strain. The virus stocks were determined to be pure of contaminating agents, and endotoxin levels were <0.23 EU/mL. The replication kinetic experiments of each strain were essentially equivalent and were comparable to the VEEV TrD strain. Based on the slightly higher titers in the prepared master and working virus stocks, the VEEV INH-9813 strain was ultimately selected.

### 3.2. Preparation of Challenge Material

The objective of the characterization of the VEEV challenge material from the virus stocks was to maintain purity, identity, potency, and uniformity of lots. The alphavirus workshop reviewed the pros and cons of three methodologies in regards to the generation of virus stocks and challenge material that would result in the reproducibility of potency (Table 3). The use of plaque-purified virus stocks carried the risk of not being able to replicate the performance of wild-type (uncloned) viral stocks if the selected plaque had suboptimal animal model fitness. The majority of experts felt that the cDNA clone-derived stock offered the most controlled pathway, as it would result in an unlimited supply of the virus derived from a well-characterized consensus sequence (expected to produce similar-sized plaques) and would avoid the potential for phenotypic changes associated with additional passages needed for the propagation of new master and working challenge material banks. Potential benefits from using uncloned (wild-type) VEEV to generate virus stocks were outweighed by identified disadvantages that included the lack of control of cell culture-adaptive mutations due to various passages, in vivo virus attenuation, and the potential need for additional culture passages to replenish the master and working challenge banks.

**Figure 1:**
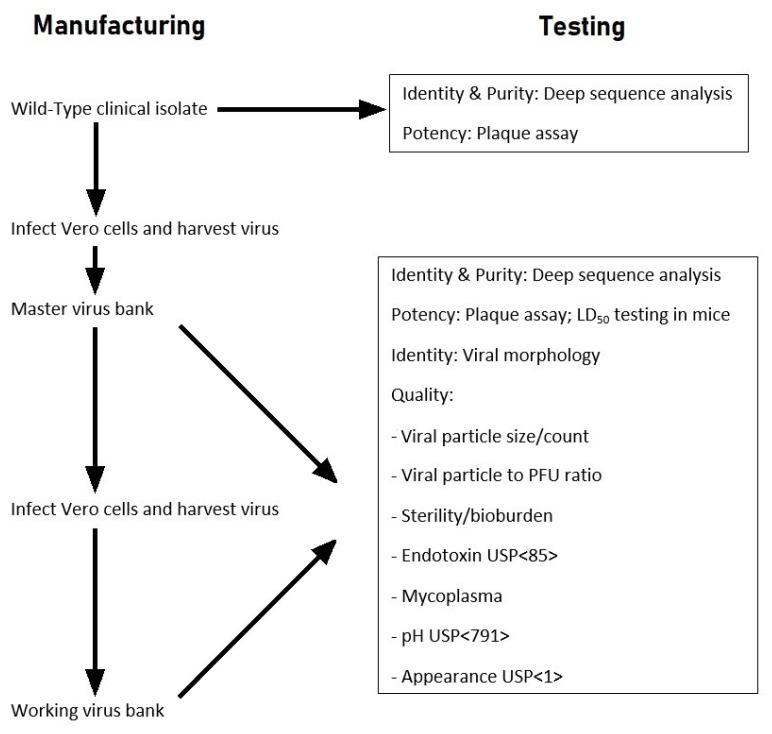
Process flow diagram for initial assessment and manufacturing uncloned (wild-type) VEEV TrD and INH 9813 strains for challenge material.

**Figure 2:**
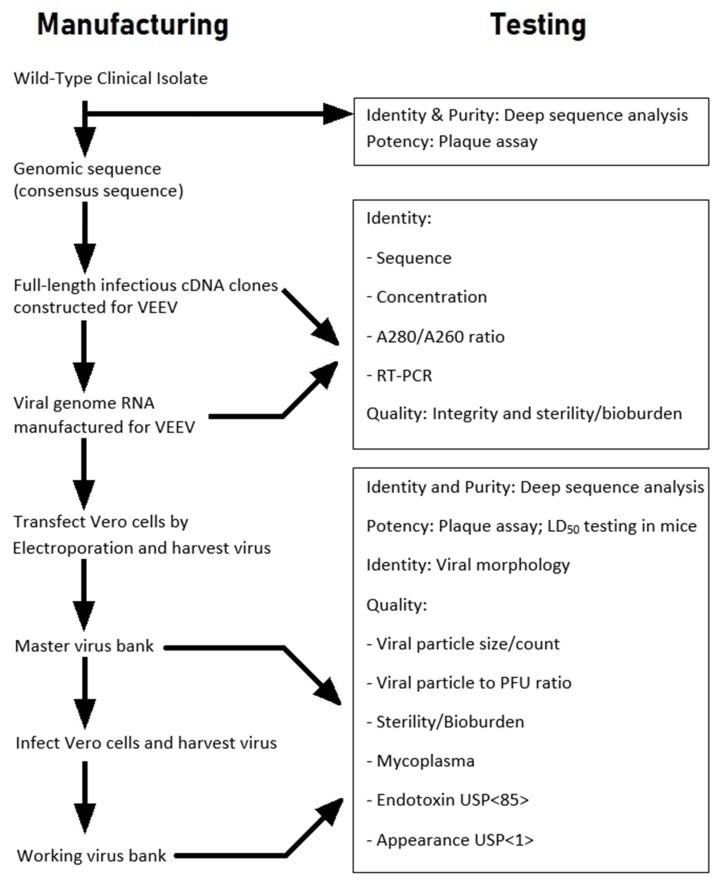
Process flow diagram for initial assessment and manufacturing cloned (cDNA) VEEV TrD and INH 9813 strains for challenge material.

A strategy was developed during the workshop to manufacture virus stocks derived from a cDNA clone of the selected VEEV strain, and to then compare the clone-derived virus to the wild-type VEEV isolate in regards to plaque size, in vitro replication kinetics in cell culture, and in vivo virulence (mouse LD_50_) (Figure 1 and Figure 2). The prepared virus stocks (master and working virus banks) would be prepared in ATCC Vero E6 cells and subjected to deep sequence analysis to characterize the virus population and consensus sequences and purity of the preparations. The use of cDNA clones would depend on the characteristics and virulence of the rescued virus being similar to the wild-type VEEV strain. If the cDNA clone characteristics were noted to be similar to the wild-type VEEV strain, a comparison of ID_50_ and targeted pathology (e.g., brain) of the cDNA clone to the wild-type VEEV isolate in NHPs would be considered. 

### 3.3. VEE Disease and Pathophysiology in Humans

#### 3.3.1. Background of VEE Disease

The literature has reported clinical manifestations of VEE to be similar in humans infected with VEEV varieties IAB, IC, ID, and IE, regardless of exposure route [51,55,56,57,58,59,60]. A recently published retrospective review of detailed medical records of laboratory- and vaccine-acquired VEEV IAB (TrD strain) infections during the U.S. Biowarfare (BW) program noted both aerosol and percutaneous routes of VEEV exposure to result in a self-limited, acute febrile illness that presented initially with an abrupt onset of fever, chills, severe headache, malaise, fatigue, weakness, back pain, myalgia (often in the lower back, thigh, or calf muscles), sore throat, anorexia, and nausea [52]. Incubation periods by both routes were similar (generally 1 to 4 days; range 2 h to 8 days), with most signs and symptoms occurring on the initial day of illness. Common physical examination findings included fever, pharyngeal erythema, conjunctival injection, and lymphadenopathy. Unlike mice and NHPs, in which aerosol-acquired infection resulted in an increased severity of central nervous system (CNS) disease compared to percutaneous-acquired infection [48,52,61,62,63,64,65,66], CNS signs and symptoms in humans were infrequent regardless of the exposure route, and severe encephalitis (e.g., seizures, paralysis, or coma) was not observed in this adult population (age 21–41 years). The only significant difference noted in aerosol-acquired infection was an association of increased upper respiratory tract-related symptoms and signs that included sore throat (with or without erythema), cervical lymphadenopathy, and neck pain. There were no deaths, and symptoms generally resolved within a week, with asthenia persisting in some cases for an additional 1 or 2 weeks. Fever, viremia, and lymphopenia were common markers of disease in humans by both exposure routes. Fever was observed in nearly all cases, viremia was common on days 1–4 of illness (range day 0–7 of illness), and lymphopenia (defined as <1500 cells/mm^3^) was common early in infection (onset generally by day 1–3 of illness). The observed increase in upper respiratory tract-related findings associated with aerosol-acquired VEE in humans is supported by the mouse model in which aerosol and intranasal (IN) challenge were associated with nasal mucosa necrosis, necrotizing rhinitis, and increased viral burden in the upper respiratory tract, and by the IN challenge NHP model that detected VEEV in the cervical lymph nodes within 18 h post-challenge.

Rusnak et al. also compared the aerosol-acquired VEEV cohort from the U.S. BW Program (VEEV IAB strain TrD) to the initial 14 aerosol-acquired VEE cases reported in laboratory workers (mainly VEEV IAB Venezuela 1938 strain), to the 24 aerosol-acquired VEE cases in Russia (subtype unknown) from a single-source laboratory exposure, and to mosquito-borne VEEV IC and ID cohorts [36,52,53]. Regardless of the exposure route or VEEV subtype, infection in adults generally presented with a self-limited febrile illness that uncommonly resulted in severe encephalitis (e.g., seizures, coma, cranial nerve abnormalities, or paralysis) at exposure doses encountered from laboratory accidents, early VEEV vaccine candidates, and mosquito bites. While aerosol-acquired VEE in humans from VEEV IC strains has not been reported, the similar clinical presentation of percutaneous-acquired VEE due to VEEV IC and VEEV IAB strains supports aerosol-acquired VEE from VEEV IAB and IC strains to also have a similar clinical presentation.

Of note, the VEEV exposure doses in humans, whether laboratory-, vaccine-, or mosquito-acquired, were likely lower than aerosol challenge doses of NHPs which often were ≥1 × 10^6^ pfu. The infrequent occurrence of CNS disease in adult humans by aerosol exposure may be species-related, with younger age and elderly being the major risk factors for CNS disease in humans. It is unknown if higher aerosol challenge doses in humans would result in increased CNS disease. 

#### 3.3.2. VEEV IAB (TrD) Infection

While the VEEV TrD outbreak (1943–44) mainly affected equids, two deaths in humans were attributed to a VEEV TrD strain, diagnosed by either viral isolation or cross-immunity testing in brain tissue. In addition, three laboratory technicians and three entomology workers developed nonlethal VEE infection due to occupational exposure [2,11,19,36,67]. Subsequent to the Trinidad outbreak, VEEV infection in humans from the TrD strain has been reported in laboratory workers after exposure to contaminated needles or infectious aerosols, and also after VEEV vaccination due to incomplete inactivation of the initial formalin-inactivated VEEV (TrD strain) vaccine administered during the U.S. BW Program [19,52,68], with exposure by both routes resulting in a similar self-limited febrile illness [52]. 

#### 3.3.3. VEEV IC (INH-9813) Infection

The VEEV IC INH-9813 strain was isolated from human serum during the 1995 outbreak in Venezuela and Colombia that resulted in 75,000 to 100,000 human cases [6,23,69]. The clinical presentation most commonly manifested as a febrile illness of 3 to 4 days duration, presenting with an acute onset of fever, chills, severe headache, myalgia, prostration, vomiting, and sometimes diarrhea. The case-fatality rate during the 1995 outbreak was estimated to be 0.7% based on random surveys of residents in the Manaure municipality of La Guajira state, Colombia, with a conservative estimate of 300 VEE-associated deaths in La Guajira state alone during the outbreak. Neurological manifestations of encephalitis included disorientation, drowsiness, mental depression, and seizures. Thirteen VEEV human isolates from this epidemic characterized antigenically and/or genetically were determined to be closely related to VEEV IC isolates from the 1962–1964 Venezuela outbreak and a 1983 mosquito Panaquire isolate from north central Venezuela [23]. No outbreaks of VEEV IC have been reported since 1995. 

Aerosol-acquired VEE due to subtype IC has not been reported in humans, and CNS histopathology in humans is limited mainly to mosquito-borne VEEV IC cases (strain unknown) (Table 4 and Table 5) [23,70]. Based on the similar clinical presentation of mosquito-borne disease from VEEV IAB and IC strains, the symptomatology of aerosol-acquired disease and CNS histopathology are also likely to be similar between the two VEEV epidemic varieties [5,52].

### 3.4. Animal Models

#### 3.4.1. VEEV Mouse Model

VEEV aerosol and IN challenge of several mouse strains (CD-1, BALB/c, outbred ICR, and C3H/HeN mice) in the literature were demonstrated to be lethal models, with death mainly due to encephalitis. Mice were challenged most commonly with wild-type VEE TrD strain or V3000 strain (VEEV derived from a cDNA clone of the VEE TrD strain with a passage history of once in guinea pig brain and 14 times in chick embryonated eggs) [48,52,61,63,72,73,74,75,76,77]. These studies demonstrated that, regardless of the exposure route, VEEV in mice entered the CNS mainly via the olfactory system due to the increased susceptibility of olfactory neurons to VEEV infection. Unlike subcutaneous (SC) challenge of VEEV that required a viremia before infection of the olfactory system, aerosol and IN challenge resulted in direct infection of the nasal mucosa and olfactory system with early neuroinvasion that occurred before the onset of viremia. 

Aerosol and IN VEEV challenge were associated with increased histopathological findings and viral burden in the upper respiratory tract, nasal mucosa, and CNS compared to parenteral challenge. Aerosol and IN challenge resulted in necrotizing rhinitis, massive infection of the olfactory epithelium, and bilateral infection of the olfactory nerves, bulbs and tracts, with CNS infection noted between 16 and 48 h post-challenge. Viral levels were observed to be three times higher in the olfactory bulb than the brain at 16 to 24 h post-aerosol challenge but were similar to viral levels in the brain at 60 h, supporting virus entry into the brain via the olfactory system. Aerosol challenge also resulted in detectable virus in the lungs within 12 h post-challenge, with subsequent viremia and viral spread to lymphoid tissues [48,52,61,62,63,78].

#### 3.4.2. VEEV NHP Model

Rhesus (*Macaca mulatta*) and cynomolgus (*Macaca fascicularis*) macaques in the literature have been assessed to be nonlethal models of VEEV infection with VEEV varieties IAB, IC, and IE. NHPs generally had onset of fever, viremia, and lymphopenia within 1 to 3 days following VEEV aerosol or parenteral challenge [52,64,79]. While some NHPs exhibited signs of encephalitis a few days later, nearly all NHPs (similar to humans) survived infection. Also, similar to disease in humans, fever, viremia, and lymphopenia were identified as markers of infection. CNS histopathology of infected NHPs noted multifocal perivascular cuffs composed mainly of lymphocytes, gliosis, satellitosis, neuronal death, and a few microhemorrhages. 

Earlier NHP studies comparing aerosol/IN to parenteral VEEV challenge were often limited due to the absence of immunohistochemistry staining, electronmicrography, and VEEV strain characterization. Nevertheless, similar to mice, these NHP studies demonstrated earlier onset and more severe CNS disease after aerosol and IN challenge as compared to parenteral challenge. Unlike the mouse model, VEEV neuroinvasion and neurovirulence were more limited, resulting in a nonlethal infection. Similar to mice studies, studies in NHPs supported aerosol and IN challenge routes to be associated with early and direct CNS infection via the olfactory pathway, including studies that detected VEEV in the olfactory bulb within 48 h post-IN challenge (compared to 6 days after intraperitoneal challenge) and before the onset of viremia. Studies demonstrated that intratracheal challenge (bypassing the upper respiratory tract) or aerosol challenge of NHPs after surgical interruption of the olfactory tracts resulted in delayed and less severe CNS infection similar to parenteral VEEV challenge [52,64,65,66]. Although clinical and histopathological findings support both the rhesus and cynomolgus macaques as potential nonlethal animal models under the Animal Rule, most recent vaccine trials have used the cynomolgus macaque model to demonstrate vaccine efficacy against aerosol VEEV challenge [72,79,80,81].

#### 3.4.3. VEEV IAB TrD Animal Models

The pathogenesis and virulence of the VEEV TrD strain and the V3000 cDNA clone-derived virus from the TrD strain were studied in mice, hamsters, and NHPs [64,77,82]. The passage history of VEEV TrD virus stocks for the majority of animal studies conducted at USAMRIID was recorded as guinea pig—one, chick embryo—14, suckling mouse brain—one, and BHK—one or two. A summary of the host susceptibility and the clinical and pathological responses to the TrD strain is provided in Table 4. Of note, aerosol and IN challenge of mice with wild-type VEEV TrD and V3000 cDNA-cloned strains resulted in nasal mucosal necrosis, necrotizing rhinitis, an increase in viral burden in the upper respiratory tract, and an earlier and more severe CNS infection compared to percutaneous challenge [48,62,63]. NHP studies also noted an earlier onset and more severe CNS infection with aerosol and IN challenge compared to parenteral challenge routes.

#### 3.4.4. VEEV IC INH-9813 Animal Models

Animal model development with wild-type and cDNA clone versions of the VEE IC INH-9813 strain is still in the early stages [71]. Aerosol challenge of BALB/c mice with the VEE IC INH-9813 strain (10 mice/group) resulted in lethal disease at low challenge doses, with 100% lethality at 2150 pfu, 80% lethality at 195 pfu, and 20% lethality at 16-pfu challenge doses (Table 5). Histopathology studies have not yet been performed. Aerosol challenge of cynomolgus macaques (challenge doses ranging from 30 to 5.94 × 10^8^ pfu) with VEE IC INH-9813 resulted in nonlethal disease in all 11 NHPs, with a febrile illness observed even at the lowest 30-pfu challenge dose (one NHP only) (Table 5). Fever, viremia, and lymphopenia were observed as potential markers of infection.

## 4. Discussion

Selection of the viral strain and methodology for propagating viral stocks for challenge material for vaccine approval under the FDA Animal Rule is critical. The propagation of the virus must result in the uniformity of virus stocks and challenge material, and challenge of the selected animal model with the selected viral strain should result in the reproducibility of disease with a comparable morbidity/mortality as humans.

The VEEV strains selected for the aerosol-challenge animal model should “ideally” be isolates from human cases that have a low passage history in animals and cell cultures. However, the DOD requirement for a vaccine to protect against VEEV strains likely to be involved in a bioterrorism event justified the selection of a VEEV IAB TrD strain, even though a donkey brain isolate with multiple passages, as the TrD strain was the only VEEV strain weaponized. Even after multiple cell culture passages, the TrD strain still demonstrates virulence in animal models and exposed laboratory workers [52]. Furthermore, the availability of documented aerosol-acquired VEE cases in humans due to VEEV IAB (mainly TrD strain) would allow for comparison of aerosol-acquired VEEV in humans to the animal model. 

However, down-selection to the VEE IC INH-9813 strain was based mainly on the strain being a human isolate with a limited passage history. While aerosol-acquired VEEV IC infection in humans has not been documented, the similarity of VEEV IAB and IC mosquito-borne disease supports the likely similarity to aerosol-acquired VEEV IAB disease and use of aerosol-acquired VEEV IAB human disease for comparison to the VEEV IC animal model. Initial VEEV INH-9813 aerosol and SC challenge studies in mice and NHPs demonstrated a similar morbidity and mortality as observed with the TrD strain. Based on the available data, the FDA concurred that the animal study data supported proceeding with the evaluation of both the TrD and INH-9813 strains in BALB/c mouse and cynomolgus macaque animal models. 

A concern in the propagation of VEEV stocks and challenge material was a potential variation in virulence associated with the different sized plaques observed in cell cultures of wild-type VEEV (both large- and small-sized plaques). Variability in plaque size was also addressed by the FANG in the generation of Ebola virus challenge material under the Animal Rule, as the larger-sized plaques of 8U Ebola (attributed to virus adaptation in cell culture) were less virulent than the smaller-sized 7U Ebola virus plaques [9]. To address and avoid variations in the virulence of viral stocks due to 7U and 8U Ebola virus plaques, the Filovirus group developed a characterization, release, and stability test plan that would be used to evaluate master and working virus stocks generated using uncloned (wild-type) methodology and the cDNA-cloned methodology. However, the propagation of virus stocks using the cDNA-cloned methodology for Ebola virus was demonstrated not to be a viable option due to non-reproducibility of lots (different consensus sequences in each lot produced) and to the lower virulence of the cDNA clone-derived virus compared to the parent clone. 

Unlike the experience of the Filovirus group, the use of the cDNA-cloned methodology to generate VEEV stocks was supported in the literature. VEEV cDNA clones derived from wild-type VEEV IC SH3 and VEEV ID ZPC728 strains exhibited in vitro and in vivo characteristics indistinguishable from their parent viruses and demonstrated similar replication kinetics as their parent virus in Vero76 and L929 cell lines [10]. SC VEEV challenge (1000 pfu) of adult National Institute of Health (NIH) Swiss mice with the cDNA-cloned strains resulted in comparable serum viral titers and time to death as their respective parent virus, and in high viremia titers within 24 h that were nearly identical to their parent strain. Disease from all four strains was manifested by decreased activity and hunching by day 3 to 4 post-infection; followed by onset of anorexia, lethargy, and hind limb paralysis by day 4 to 7 that progressed to stupor and coma; and then death due to encephalitis on day 7 or 8. A VEEV ID ZPC738 cDNA clone was also developed and demonstrated to be phenotypically indistinguishable from its enzootic parent strain [11]. Also, a VEEV IAB V3000 cDNA mutant clone (a single I239N mutation with a passage history in guinea pigs once and 14 times in embryonated eggs) derived from wild-type TrD demonstrated a similar LD_50_ as its parent wild-type TrD strain in aerosol-challenge studies in 4-week-old female CD-1 mice, as did a recently produced VEEV IAB V3000 cDNA clone (using similar methodology) without the I239N mutation [74,77,83]. The original V3000 cDNA clone resulted in a similar lethal illness in mice and nonlethal febrile illness with viremia in NHPs as reported with wild-type VEEV TrD and was also used to evaluate the mechanism of neuroinvasion in mice and to assess vaccine efficacy in NHPs [61,63,72,82]. Lastly, initial comparison of the virulence of a newly developed cDNA clone of VEEV TrD GenBank reference sequence L01442.2 to wild-type VEEV TrD (passage history in guinea pigs once, chick embryos 13 times, and duck embryo cells once) showed similar lethality in mice following aerosol challenge, with 100% mortality after challenge with 7 pfu of cDNA-cloned VEEV TrD or 28 pfu of wild-type VEEV TrD [84]. Only the 4-pfu aerosol challenge dose with wild-type VEEV TrD resulted in survival of some mice (estimated LD_50_ of 4.6 pfu). 

Based on the experience with cDNA-cloned stocks and the identified risks in generating virus stocks using wild-type or plaque-cloned viruses, the cDNA-cloned methodology was chosen as the path forward to propose to the FDA for the generation of virus stocks and challenge material. The cDNA-cloned methodology would provide a more controllable path with use of a clear consensus sequence (verified by sequencing of the original population), avoid multiple major variants, and result in an unlimited supply of the virus without additional passages. The use of cDNA clones hypothetically has the potential to result in a reduction in single nucleotide polymorphism (SNP) diversity as compared to the wild-type parenteral stock. And though reduction in SNP diversity has been associated with high viral fidelity variants that are also attenuated, animal studies indicate that clone-derived VEEV stocks are as virulent if not more virulent than their originating parent (uncloned) stock [10,11,83,84,85,86].

Subsequent communication with the FDA resulted in their concurrence on the usage of the cDNA-cloned methodology to generate viral stocks, provided the virulence of the cDNA-cloned VEEV stocks was comparable or noninferior to the uncloned (wild-type) VEEV stocks as outlined in Figure 1 and Figure 2. Comparison of ID_50_ and targeted pathology (e.g., brain) of the cDNA clone to the wild-type VEEV strain in NHPs would be considered if the cDNA clone characteristics were similar to the wild-type VEEV isolate. After the generation of sufficient data, the overall characterization plan would be reviewed and modified to a more efficient approach as assays are refined and the master and working viral stocks are characterized.

Vaccine licensure under the Animal Rule for the DOD VEEV vaccine initially will be requested only for protection against aerosol challenge with VEEV TrD strain. However, VEEV vaccine candidates demonstrating protection against the TrD strain will likely protect against other IAB and IC epizootic strains, based on phylogenetic/antigenic similarities among the epizootic strains [31,39,46,78,87]. VEEV IAB strains showed only a 96.1 to 99.2% genetic distance between strains on full sequence analysis [31,88]. Only a 15 amino acid difference was observed in the E2 glycoprotein between a VEEV IC epizootic strain derived from a VEEV ID enzootic strain following site-directed mutations in the E2 glycoprotein [11,88]. While phylogenetic differences may be greater with VEEV IE and the former VEEV complex subtypes VEE IF and VEE complex subtypes II to IV, the highly conserved E1 protein among alphaviruses may potentially result in cross-immunogenicity of epizootic-based vaccines against these former VEE complex subtypes via vaccine-induced antibodies to E1 and E2 envelope proteins [88,89,90,91].

Vaccine cross-protection from VEEV IAB strain-derived vaccines is supported by serological testing and/or animal challenge studies that demonstrated immunogenic responses and/or cross-protection against other VEEV IAB strains, other subtype I varieties, and/or former VEE complex subtype II and IIIA viruses. The VEEV TC-83 investigational vaccine, a live, attenuated vaccine derived from the VEEV IAB TrD strain, resulted in long-term (9 years) persistence of plaque reduction neutralization antibodies in humans against VEEV IC strain V-198 but not against enzootic VEE IE [80,85]. However, a more immunogenic, live-attenuated V3526 vaccine candidate (also derived from VEEV TrD strain), protected NHPs against VEEV IE (68U201) aerosol challenge even though it failed to elicit measurable neutralizing antibodies to VEEV IE in six of eight NHPs [80,90]. A virus-like replicon particle (VRP) VEEV vaccine candidate demonstrated long-term (12 months) cross-protection in mice (100% survival) against aerosol challenge to VEEV IAB, VEEV IE, and Mucambo virus (former VEEV subtype IIIA), that was associated with a strong VEEV-specific IgG-specific ELISA response but a minimal neutralizing antibody response [39]. VRP vaccination in all NHPs was associated with a measurable neutralizing antibody response to VEE IAB (TrD), VEEV IC (p676 strain), VEEV ID (3880 strain), and VEEV IE (68U201 strain). Lastly, cross-protection against IE and former VEEV subtypes was demonstrated with a humanized monoclonal antibody (Hu1A3B-7) derived from bone marrow donors immunized with TC-83, that protected mice against aerosol challenge to VEEV TrD, VEEV IE, Mucambo virus (former VEE complex subtype IIIA), and Everglades virus (former VEE complex subtype II) [42]. 

Clinical presentation of VEEV IAB-induced disease in humans by both aerosol and percutaneous exposure routes has been well characterized [52]. Unlike mice and NHPs, an increased severity of encephalitis was not observed with aerosol-acquired VEE in humans. Nevertheless, a greater disease severity in mice and NHPs does not preclude their use as animal models, if the models are stricter than human disease.

## 5. Conclusions

Systematic approaches were developed for virus strain selection and selection of the optimal methodology for the propagation of viral stocks and challenge material for the animal models, as required for VEEV vaccine approval under the FDA Animal Rule. The VEEV IAB TrD and IC INH-9813 strains were selected for vaccine development challenge strains in the BALB/c mouse and cynomolgus macaque animal models. The selection of cDNA-clone methodology to generate virus stocks and challenge material was based on the use of a stable consensus sequence being a more controlled path, providing an increased likelihood of uniformity of lots and an unlimited virus supply without additional passages. These methodologies may serve as a template for strain selection for other vaccine programs using the Animal Rule. 

## Figures and Tables

**Table 1:** Venezuelan equine encephalitis virus (VEEV) strain selection criteria for VEE vaccine animal model [7,8].

Source	Strain Selection Criteria
**Department of Defense (DOD)**	Strains that will support Food and Drug Administration (FDA) licensure for vaccine protection against at least two VEEV subtype I variants
**DOD**	Strains relevant to a bioterrorism event
**FDA**	Strains isolated from lethal human cases or associated with causing human disease
**FDA**	Strains with known and low passage history
**Filovirus Animal Non-clinical Group (FANG)**	Strains with no passage history in animals (if available)
	Strains with low passage history in cell culture
**FDA**	Strains that mirror the expected disease state in humans
**Logistics**	Strains available and accessible to laboratories licensed to work with select agents
**Logistics**	Ability to grow strain to yield sufficient challenge material for animal model

**Table 2:** VEEV epizootic (IAB, IC) and enzootic (ID, IE) strains.

Strain Type	Strain	VEEV Subtype ^i)^	Source	Passage History	Place and Year of Isolation	Reference
**Epizootic VEEV Strains**	Trinidad Donkey (USAMRIID)	IAB	Donkey brain	GP1, CE14, SMB1, BHK-1 or 2	Trinidad, 1943	[17,18,19,20]
	Trinidad Donkey (USAMRIID)	IAB	Donkey brain	GP1, CE13, DE cells 1		
	Trinidad Donkey (CDC)	IAB	Donkey brain	GP1, V6, BHK1	Trinidad, 1943	[21,22]
	69Z1	IAB	Human	SM2, V1	Guatemala, 1969	[20]
	69Z1	IAB	Human	BHK1, unknown	Guatemala, 1969	[20]
	INH-9813	IC	Human serum	V1	Venezuela, 1995	[23]
	INH-6803	IC	Human serum	V1	Venezuela, 1995	[23]
	SH3	IC	Human	V1	Venezuela, 1993	[10,24]
	3908	IC	Human serum	C6/36-1	Venezuela, 1995	[10,15,23,25]
	6119	IC	Human serum	BHK1	Venezuela, 1995	[20,23,26]
	V198	IC	Human serum	SM1, V1, DE1	Colombia, 1962	[26]
	V178	IC	Horse brain	SMB2, V3	Colombia, 1961	[23]
	P676	IC	*Aedes triannulatus*	SM1, V3, BHK1	Venezuela, 1963	[26]
**Enzootic VEEV Strains**	3880	ID	Human	SM3, V4, BHK1	Panama, 1961	[20,27,28]
	FSL0201	ID	Human serum	V1	Peru, 2000	[29]
	83U434	ID	Hamster	CE cells 1, V1, BHK1	Colombia, 1983	[13,23]
	An9004	ID	Hamster	SM3, V1, BHK1	Colombia, 1969	[20,27]
	66637	ID	Hamster	SM1, V1	Venezuela, 1981	[13,20]
	306425	ID	Hamster	Unknown, BHK1	Colombia, 1972	[20]
	ZPC738	ID	Hamster	Unknown, BHK1	Venezuela, 1997	[13]
	V209A	ID	Mouse	SM2, V2	Colombia, 1960	[20,27]
	68U201	IE	Hamster	SM1, BHK2, CE cells 3	Guatemala, 1968	[30]
	93-42124	IE	Horse (brain)	SM1, CE cells 1	Chiapas, Mexico, 1993	[30]
	CPA-201	IE	Horse	SMB1, RK1, BHK2	Chiapas, Mexico, 1993	[30]
	96-32863	IE	Horse (brain)	SM1, CE1	Oaxaca, Mexico, 1996	[30]

NA = not available. BHK, baby hamster kidney cells; C6/36, mosquito cell line; CE, chick embryo; DE, duck embryo; GP, guinea pig; RK, rabbit kidney cells; SM, suckling mouse; SMB, suckling mouse brain; V, Vero cells; USAMRIID, U.S. Army Medical Research Institute of Infectious Diseases.

**Table 3:** Comparison of pros and cons for three methods of the propagation of VEEV stocks.

Population Type	Pros	Cons
**Uncloned (wild-type)**	- Maintenance of wild-type diversity	- Cell culture adaptation often results in artificial amino acid substitutions and in vivo attenuation - Risk that multiple major variants present in the population can confound identification of genetic determinants of important phenotypes - Limited supply without additional passages
**Plaque-cloned**	- Clear consensus sequence (verified by sequencing original population) and lack of multiple variants	- Possible reduced single nucleotide polymorphism diversity - Risk of selection of a suboptimal fitness mutant (change in the consensus and master sequence) - Limited supply without additional passages
**cDNA-cloned**	- Clear consensus sequence (verified by sequencing the first-generation population) and lack of multiple major variants - Unlimited supply of the same master virus without passages	- Possible reduction in single nucleotide polymorphism diversity

**Table 4:** Summary of the selected VEEV IAB TrD strain in mice, NHPs, and humans.

	BALB/c Mouse	Cynomolgus Macaque	Humans
**Route of Exposure**	SC (scruff of neck, footpad) Aerosol (whole body)	Aerosol	Aerosol (laboratory exposure) Parenteral (vector-borne, laboratory exposure, vaccine-related)
**Disease and Mortality**	Lethal infection after SC and aerosol exposure due to encephalitis even at low challenge doses, with death 5–7 days post-challenge [48,64]	Generally non-lethal infection after SC and aerosol exposure at high challenge doses (100% infection after aerosol challenge 1 × 10^8^ pfu); death uncommon. Increased severity of CNS disease after aerosol challenge [52,64].	Generally non-lethal infection after SC or aerosol exposure [52] Infection common after exposure to low doses. Encephalitis uncommon (<0.5%); mainly in children/elderly
**Exposure Dose**	LD_50_ SC ~10 pfu; LD_50_ aerosol <1 pfu ^a^	ID_50_ unknown	Low infective dose (exposure dose unknown in humans)
**Disease Onset**	24–72 h	24–48 h	24–72 h (range 24 h–8 days)
**Signs and Symptoms; Progression of Disease**	Nearly 100% mice infected develop disease. Mice infected by the SC route demonstrated initial signs of decreased grooming and ruffled fur 2–3 days after challenge; followed by lethargy, hunched posture, and hind-limb paralysis; death or euthanasia 5–7 days after challenge. Mice infected by aerosol route demonstrated onset of similar signs 2–3 days after challenge, and death or euthanasia 6–7 days after challenge.	Aerosol VEEV TrD challenge results in fever and lethargy within 24–48 h after challenge. Fever resolved by D9. May have mild tremors. Generally NHPs have full recovery.	Self-limiting febrile illness, usually <1 week duration (asthenia may persist 1–2 weeks). Symptoms of high fever, chills, severe headache, back pain, malaise, myalgia, anorexia, nausea, sore throat, fatigue, photophobia, and/or vomiting. Encephalitis uncommon (<1% cases); manifested by decreased sensorium, confusion, gait abnormalities; severe cases with seizures, paralysis, or coma. Most cases recover without neurological sequelae.
**Clinical Laboratory**	Not done.	Viremia observed initially on D1–D2 that resolved by D3–D4 Lymphopenia observed early in illness. Increase in WBC observed later in some NHPs [37]	Viremia common D1–D4 (range D1–D7 illness) after aerosol and SC challenge. VEEV isolation from pharynx common D1–D4 (range D1–D7) in VEE IAB TrD and VEE IAB Co1938 strains [52]. Lymphopenia common D1–D3 of illness, with improvement after D3 and recovery by D6. Leukopenia may occur D3–D5; resolves by D5–D8 illness [52].
**Pathology**	Death due to encephalitis. CNS entry occurs via the olfactory system. Histological lesions present in both neural and extraneural tissues; CNS lesions characterized by necrotizing panencephalitis and myelitis; congestion and minor hemorrhage, damaged endothelial cells, perivascular edema, minimal necrosis and infiltration of a few neutrophils and mononuclear cells (no vasculitis) [48,64]	Limited pathology studies in cynomolgus macaques with TrD strain. Intraperitoneal challenge of Rhesus macaques showed histopathology findings initially in lymphoid tissues; lesions in olfactory cortex and thalamus by D6, and in hypothalamus and throughout brain by D8. Predominant lesions of gliosis and multifocal perivascular cuffing composed of lymphocytes. CNS lesions most severe between D14 and D21 post-challenge (observed in 18/20 NHPs). Aerosol challenge rhesus macaques (VEEV-subtype unknown) noted VEEV in nasal mucosa, lungs, cervical/hilar lymph nodes by 18 h, olfactory bulb by 48 h; more severe CNS infection than intraperitoneal challenge (higher CNS viral titers, increased neuronal damage, neuronophagia, and neutrophil infiltration); CNS infection preceded onset of viremia [52,64,65,66].	CNS pathology available in humans with VEEV IC (see Table 5).

D = day; SC = subcutaneous. ^a^ Starting concentration and all-glass impinger samples for aerosol exposure quantitated by plaque assay to determine titer (pfu/mL); Guyton and Bide formulas used to calculate the inhaled exposure dose per animal.

**Table 5:** Summary of the selected VEEV IC INH-9813 strain in mice, nonhuman primates (NHPs), and humans.

	BALB/c Mouse	Cynomolgus Macaque	Humans (VEEV IC Strains)
**Route of Exposure**	SC (scruff of neck); Aerosol (whole body)	Aerosol (head only)	Parenteral (mosquito-borne). No aerosol-acquired VEEV IC cases reported
**Disease and mortality**	Lethal infection after SC and aerosol exposure due to encephalitis even at low challenge doses (100% mortality after 100 pfu challenge dose), with death or euthanasia 6–8 days after challenge [71]	Non-lethal infection (100%) in 11 NHPs after aerosol challenge (dose range 5 × 10^4^ to 5.94 × 10^8^ pfu; 30 pfu in a single NHP) [71]	1995 Colombia/Venezuela outbreak with VEE IC INH-9813 strain (mosquito-borne disease) [6,23]. Generally non-lethal infection after SC exposure. No reported cases of aerosol-acquired VEEV IC. Infection common after exposure to low doses. Encephalitis uncommon; mainly in children/elderly
**Exposure Dose**	LD_50_ SC =5 pfu; LD_50_ aerosol = 53 pfu ^a^	ID_50_ aerosol ≤ 30 pfu (*n* = 1)	Low infective dose (human infective dose unknown)
**Disease Onset**	48 h to 6 days	24 to 48 h	27.5 h to 4 days in 11 mosquito-borne VEE IC cases (strain unknown) [5]
**Clinical Manifestation**	VEEV INH-9813 resulted in infection after SC and aerosol exposure (100% infection). Mice with infection demonstrated initial signs of decreased grooming and ruffled fur 6 days after SC challenge or 48 h after aerosol challenge, followed by lethargy, hunched posture, and hind-limb paralysis (likely due to encephalitis), with death or euthanasia 6–8 days after SC or aerosol challenge	VEEV INH-9813 resulted in 100% infection after aerosol challenge. NHPs demonstrated initial signs of fever and lethargy 24–48 h after challenge, with fever resolving by D7. Biphasic fever noted only at highest dose tested. Tremors of variable duration (4–8 days; maximum 26 days) in 50% of NHPs. All NHPs survived	Mosquito-borne VEEV IC infection (strain unknown) similar to VEE IA/B infection in well-characterized outbreak in Texas (*n*-88) [5]. Self-limiting febrile illness of 1-week duration (asthenia may persist for 1–2 weeks). Symptoms included high fever, chills, severe headache, myalgias, malaise, and anorexia. Also, nausea, vomiting, sore throat, photophobia, ocular pain, arthralgia, somnolence and drowsiness reported. Encephalitis in 2% adults (mild) and 7.6% children. Encephalitis manifested by decreased sensorium, disorientation, delirium, nuchal rigidity, ataxia, seizures; coma and paralysis in severe cases
**Clinical Laboratory**	Unknown	Viremia detected starting on D1–D2 post-challenge and resolved by D3–D4. Lymphopenia typically noted early in infection. Elevated WBC count detected later in some NHPs (*n* = 6)	VEE IC outbreak (strain unknown) [5]: Viremia documented in 40 cases from D0–D8 of illness (most common on D3 of illness). Lymphopenia (<1490 cells/mm^3^) in 80% cases on D1–D4 of illness; increase generally on D4 with recovery by D9. Leukopenia (<4500 cells/mm^3^) in 75% cases at D1–D2, (recovery of total WBC by D3/neutropenia by D6–D8)
**Pathology**	Pathology studies not performed	Mild to moderate lymphocytic perivascular cuffing with gliosis in CNS at day 28 post-challenge. No viral antigen detected in CNS	Autopsy of 21 mosquito-borne VEEV IC cases (strain unknown): Cerebrovascular congestion (*n* = 14), edema with inflammatory infiltrates in brain/spinal cord (*n* = 17), intracerebral hemorrhage (*n* = 7), vasculitis (*n* = 4), meningitis (*n* = 13), encephalitis (*n* = 7), cerebritis (*n* = 5). Vasculitis, fibrin thrombi, perivascular hemorrhage and edema, occasional necrosis of blood vessel walls. Inflammatory infiltrates with lymphocytic and mononuclear cells, neutrophils, histiocytes. Lymph nodes and spleen with marked lymphoid depletion/follicular necrosis; hepatocellular degeneration and congestion (11/18 cases); interstitial pneumonia (19/21 cases) and pulmonary edema (11/21 cases) [70]

D = day; SC = subcutaneous. ^a^ Starting concentration and all-glass impinger samples for aerosol exposure quantitated by plaque assay to determine titer (pfu/mL); Guyton and Bide formulas used to calculate the inhaled exposure dose per animal.
